# Worldwide initiatives aimed to train professionals in the use of the ICD-11

**DOI:** 10.1186/s12991-021-00370-2

**Published:** 2021-11-02

**Authors:** Giulia M. Giordano

**Affiliations:** WHO Collaborating Centre for Research and Training in Mental Health, University of Campania L. Vanvitelli, Naples, Italy

**Keywords:** International Classification of Diseases, 11th edition (ICD-11), Chapter on mental disorders, Training of professionals

## Abstract

**Background:**

The chapter on mental disorders of the 11th revision of the International Classification of Diseases (ICD-11) has been now finalized. Training of mental health professionals in the use of the chapter is taking place worldwide.

**Main body of the abstract:**

Information is provided on the ICD-11 training courses taking place recently, including that co-organized by the Naples World Health Organization (WHO) Collaborating Centre on Research and Training in Mental Health and the European Psychiatric Association; those which will be held in the next few months, such as the one co-organized by the World Psychiatric Association and the Global Mental Health Academy, to be held online from 8 to 29 November 2021; and the training course set up by the WHO Collaborating Centre on Mental Health at the Columbia University, in collaboration with the WHO Department of Mental Health and Substance Use, which can be accessed only by the members of the WHO Global Clinical Practice Network.

**Conclusion:**

Psychiatrists of all countries of the world are encouraged to become familiar with the ICD-11 chapter on mental disorders, which will be adopted shortly by most countries worldwide.

## Background

The chapter on mental, behavioural and neurodevelopmental disorders of the 11th revision of the International Classification of Diseases (ICD-11), has now been completed. The structure of the chapter, the most important changes with respect to the 10th revision of the International Classification of Diseases, and the most significant differences from the Diagnostic and Statistical Manual of Mental Disorders, 5th edition, have been recently described in detail [1]. The many issues that have been addressed during the development of the chapter—including the role of a dimensional component, the question of the frequent concomitance of multiple psychiatric diagnoses, and the need of a further characterization of the individual patient after a diagnosis has been made in order to guide the formulation of the management plan—have been discussed in the literature [2–17].

Education and training of mental health professionals in the use of the ICD-11 chapter is now taking place worldwide, mostly focusing on psychiatrists and psychologists, under the supervision of a WHO International Advisory Group led by G.M. Reed. Training courses have been implemented within the 18th and 19th World Congresses of Psychiatry (Mexico City, 2018; Lisbon, 2019) [18–20].

## Main text

In this manuscript, information is provided on the ICD-11 training courses taking place recently. In April 2021, the Naples World Health Organization (WHO) Collaborating Centre on Research and Training in Mental Health and the European Psychiatric Association organized an online 20-h training course, coordinated by G.M. Reed and M. Maj. The course, subdivided into four sessions, has covered schizophrenia and other primary psychotic disorders, disorders specifically associated with stress, mood disorders, anxiety and fear-related disorders, obsessive–compulsive and related disorders, feeding and eating disorders, personality disorders, disorders due to substance use, disorders due to addictive behaviours, and compulsive sexual behaviour disorder. W. Gaebel, M. Cloitre, M. Maj, C.S. Kogan, P. Monteleone, M. Swales, J.B. Saunders and N.A. Fineberg composed the Faculty.

A similar training course, co-organized by the World Psychiatric Association and the Global Mental Health Academy, will take place online from 8 to 29 November 2021 (see www.wpanet.org). The Faculty will include W. Gaebel, M. Cloitre, M. Maj, C.S. Kogan, O. Gureje, M. Swales, J.B. Saunders and N.A. Fineberg. A training session covering psychotic disorders and mood disorders was organized by the psychiatric association of Turkey in June 2021. A similar event was organized by the UK Royal College of Psychiatrists in May 2021.

The WHO Collaborating Centre on Mental Health at the Columbia University, in collaboration with the WHO Department of Mental Health and Substance Use, has recently set up a training course with exclusive access to the members of the WHO Global Clinical Practice Network (https://gcp.network). The course consists of 15 online training units, each focusing on a different group of disorders and taking from one hour to one hour and a half. Each unit provides a description of the relevant diagnostic group and the main innovations with respect to the ICD-10. The outcome of training is checked through knowledge questions. Participants have the opportunity to practice by applying diagnostic guidelines to clinical case examples. This training course is going to be available also in Spanish, and additional translations are planned.

The WHO Global Clinical Practice Network now includes more than 16,000 clinicians from 159 countries (40% from Europe, 25% from Western Pacific, 24% from the Americas, 5% from Southeast Asia, 3% from Eastern Mediterranean, and 3% from Africa; 63% from high-income countries, 37% from middle- and low-income countries). Among them, about 51% are psychiatrists and 30% psychologists.

## Conclusions

All health professionals working in mental health or primary care are welcome to join the Network in order to become familiar with the ICD-11 chapter on mental disorders, which will be adopted shortly by most countries worldwide.

## Data Availability

Data sharing is not applicable to this article as no datasets were generated or analysed during the current study.

## Abbreviations

ICD-11: The 11th revision of the International Classification of Diseases
WHO: World Health Organization
